# Spatiotemporal Dynamics and Demographic Profiles of Imported *Plasmodium falciparum* and *Plasmodium vivax* Infections in Ontario, Canada (1990–2009)

**DOI:** 10.1371/journal.pone.0076208

**Published:** 2013-09-30

**Authors:** Mark P. Nelder, Curtis Russell, Dawn Williams, Karen Johnson, Lennon Li, Stacey L. Baker, Sean Marshall, Wendy Bhanich-Supapol, Dylan R. Pillai, Filip Ralevski

**Affiliations:** 1 Enteric, Zoonotic and Vector-borne Diseases, Communicable Disease Prevention and Control, Public Health Ontario, Toronto, Ontario, Canada; 2 Surveillance Services, Communicable Disease Prevention and Control, Public Health Ontario, Toronto, Ontario, Canada; 3 Analytic Services, Knowledge Services, Public Health Ontario, Toronto, Ontario, Canada; 4 Ministry of Environment, Toronto, Ontario, Canada; 5 Department of Medicine, McMaster University, Hamilton, Ontario, Canada; 6 Department of Microbiology, Immunology, and Infectious Diseases, University of Calgary, Calgary, Alberta, Canada; 7 Parasitology, Public Health Ontario Laboratory - Toronto, Public Health Ontario, Toronto, Ontario, Canada; Johns Hopkins Bloomberg School of Public Health, United States of America

## Abstract

We examined malaria cases reported to Ontario’s public health surveillance systems from 1990 through 2009 to determine how temporal scale (longitudinal, seasonal), spatial scale (provincial, health unit), and demography (gender, age) contribute to *Plasmodium* infection in Ontario travellers. Our retrospective study included 4,551 confirmed cases of imported malaria reported throughout Ontario, with additional analysis at the local health unit level (i.e., Ottawa, Peel, and Toronto). During the 20-year period, *Plasmodium vivax* accounted for 50.6% of all cases, *P. falciparum* (38.6%), *Plasmodium* sp. (6.0%), *P. ovale* (3.1%), and *P. malariae* (1.8%). During the first ten years of the study (1990–1999), *P. vivax* (64% of all cases) was the dominant agent, followed by *P. falciparum* (28%); however, during the second ten years (2000–2009) the situation reversed and *P. falciparum* (55%) dominated, followed by *P. vivax* (30%). The prevalence of *P. falciparum* and *P. vivax* cases varied spatially (e.g., *P. falciparum* more prevalent in Toronto, *P. vivax* more prevalent in Peel), temporally (e.g. *P. falciparum* incidence increased during the 20-year study), and demographically (e.g. preponderance of male cases). Infection rates per 100,000 international travellers were estimated: rates of infection were 2× higher in males compared to females; rates associated with travel to Africa were 37× higher compared to travel to Asia and 126× higher compared to travel to the Americas; rates of infection were 2.3–3.5× higher in June and July compared to October through March; and rates of infection were highest in those 65–69 years old. Where exposure country was reported, 71% of *P. falciparum* cases reported exposure in Ghana or Nigeria and 63% of *P. vivax* cases reported exposure in India. Our study provides insights toward improving pre-travel programs for Ontarians visiting malaria-endemic regions and underscores the changing epidemiology of imported malaria in the province.

## Introduction

The annual burden of malaria, estimated at 216 million cases and more than 600,000 deaths, is far reaching in present-day tropical and subtropical climes [1]. Historically, malaria extended beyond the tropics into temperate Asia, Europe, and North America, including Canada. Malaria shaped Canada’s early history and nation building, contributing to the morbidity and mortality of early settlers. For instance, malaria exacted a toll on workers constructing the Rideau Canal (1826–1832), a vital waterway linking the Ontario cities of Ottawa and Kingston; 50% of workers were infected (putative agent was *Plasmodium vivax*) and 4% of those infected died [2]. While autochthonous *Plasmodium* transmission is absent, approximately 500 malaria cases are diagnosed in Canada every year, with the majority of these occurring in Ontario [3]. Malaria continues to affect public health outside its precinctive boundaries and presents challenges to prevention, diagnostics, and treatment programs in non-malaria-endemic regions.

Increased international travel and climate change are among the causes often cited for the increased threat and emergence of imported malaria. Advances in air travel have contributed to the swell in international travel, with 940 million international travellers in 2010 compared with 25 million in 1950 [4]. Annually, international travel to malarial zones has increased to 80-90 million travellers and has contributed to at least 30,000 imported malaria cases worldwide [5,6]. In Canada, 60% of new immigrants settle in Ontario, providing the inherent link between Ontario’s residents and malarial zones [7]. Notwithstanding impacts in malaria-endemic regions, climate change in Ontario presents the risk of local *Plasmodium* transmission via imported malaria. In densely populated regions of southern Ontario, modelling predicts the number of consecutive days above 18°C will increase from less than 1 month to 3 months in the coming decades, conditions conducive to the development of the local vector, *Anopheles* *quadrimaculatus* *sensu lato* Say, and the parasites, *Plasmodium* spp. [8]. Coupled with climate change, international travel will increase the risk of imported malaria, making malaria surveillance an important pillar of public health protection.

The primary goals of malaria surveillance in non-malarial jurisdictions, such as Ontario, are to: 1) better understand the demographics of cases and risk factors associated with infection, leading to improvement of prevention programs, educational campaigns, and policies; and 2) monitor for spatiotemporal clusters signalling the potential for local transmission. We examined malaria cases reported to Ontario’s public health surveillance systems from 1990 through 2009 to determine how temporal scale (longitudinal and seasonal trends), spatial scale (provincial, health unit diagnosing imported malaria case, exposure country), and demography (gender, age) contribute to *Plasmodium* infection in Ontario travellers. The imported malaria trends reported here will aide in the improvement of pre-travel programs and risk assessments for those travelling to malaria-endemic regions.

## Materials and Methods

### Study Location and Population

Ontario is the most populous province in Canada (13.4 million) and accounts for approximately one third of Canada’s population; more than 85% of Ontario’s residents live in urban centers, with the largest concentration located at the southwestern end of Lake Ontario [9]. In Ontario, 36 local health units administer public health services including reportable disease surveillance. Additional analyses will focus on three health units, specifically The City of Toronto Health Unit (herein Toronto; population 2,500,000), The Regional Municipality of Peel Health Unit (Peel; 1,160,000), and The City of Ottawa Health Unit (Ottawa; 800,000).

### Surveillance Systems and Ethics

Malaria is a reportable disease in Ontario through the integrated Pubic Health Information System (iPHIS), a centralized, web-based, passive surveillance information system for case reporting and management. In 2005, iPHIS replaced the Reportable Disease Information System (RDIS), with conversion of specific RDIS data fields by the end of 2005. Both systems capture demographic, clinical, and exposure data on reported cases. For this study, confirmed malaria cases reported from 1990 through 2009 were extracted from iPHIS for analyses. Exposure analyses were restricted to cases reported from 2006–2009 because exposure data from RDIS were not converted to iPHIS. Exposure data is missing or unknown for 43% of the cases from 2006 through 2009; therefore, results from analysis of exposures need to be interpreted with caution. Raw data for total imported malaria cases (*P. vivax* and *P. falciparum* only) was reported previously for Ontario (1990–2002) for a national study [3]; however, this surveillance period is included here because we provide additional data and analyses (e.g., gender, age, exposure country, health unit-level incidence).

This manuscript reports on surveillance activities and not research, and therefore research ethics committee approval was not required. Public Health Ontario (PHO) has a legislated mandate “to develop, collect, use, analyse and disclose data, including population health, surveillance and epidemiological data, across sectors, including human health… in a manner that informs and enhances healthy public policy and public health planning, evaluation and action” (Ontario Agency for Health Protection and Promotion Act, SO 2007, c 10. Available at: http://www.e-laws.gov.on.ca/html/statutes/english/elaws_statutes_07o10_e.htm). The collection, use, analysis, and disclosure of data described in the current manuscript fall entirely within this surveillance mandate. No additional data were collected, and no data outside of our mandated jurisdiction were included. Patient consent was waived as the research involved retrospective analyses of aggregated, de-identified data.

### Case Definition and Confirmation

To be considered a confirmed case in Ontario, there must be laboratory validation (demonstration of *Plasmodium* sp. in a blood smear/film) of infection with or without clinically compatible signs and symptoms [10]. Incident cases were defined as: 1) an individual’s first attack of malaria in Canada, regardless of the occurrence of previous attacks outside of the country, 2) a subsequent attack in the same person caused by a different *Plasmodium* species, or 3) a repeat attack by the same *Plasmodium* species in an individual that traveled to a malaria-endemic area after their first attack [10].

For diagnosis, blood was collected and stored in an EDTA tube and typically, a commercially-available antigen test (used since 2007; BinaxNOW Malaria Test, Binax Inc., Scarborough, ON) was performed to determine presumptive *Plasmodium* infection (test differentiates between *P. falciparum* and other *Plasmodium* species, but will not speciate *P. malariae*, *P. ovale*, and *P. vivax*). Light microscopy was used to detect Giemsa-stained parasites inside of erythrocytes with traditional thick and thin blood films [11]. Before confirming a negative result, 200 oil immersion visual fields at a magnification of 1000× were examined on both thick and thin smears. Since 2009, in cases where the thick and thin film was insufficient to detect or speciate the parasite, real-time PCR was conducted [12]. Our work includes *Plasmodium* sp., a designation representing instances where existing diagnostic tools could not differentiate between two or more *Plasmodium* species.

### Estimated Infection Rates and Statistical Analyses

In order to better assess risk for travellers, we estimated rates of infection per 100,000 international travellers based upon available international travel data. For yearly and monthly infection rates, rates per 100,000 international travellers were estimated based on total Canadians travelling to international locales (except USA) and returning to Ontario (1990–2009) [13]. For infection rates in males and females, rates per 100,000 international travellers were estimated based on total Canadians returning to Ontario from international locales (except USA) (1995–2009) [14]. For infection rates according to age group, rates per 100,000 international travellers were estimated based upon total non-permanent residents travelling to Ontario (1990–2009) [15]. The five following groups are referred to as non-permanent residents: (1) people residing in Canada claiming refugee status; (2) people residing in Canada who hold a study permit; (3) people residing in Canada who hold a work permit; (4) people residing in Canada who hold a Minister’s permit; (5) all non-Canadian born dependents of people claiming refugee status, or of people holding study permits, work permits or Minister’s permits and living in Canada [15]. For infection rates according region of travel, rates per 100,000 international travellers were estimated based on total number of non-permanent residents travelling to Ontario from each region (2006–2009) [16]. It is important to note that these rates are crude estimates and not all international travellers (except USA) visit a malaria-endemic country; therefore, our estimated rates for travellers are likely an under-estimate of the true rate of infection and results should be interpreted with caution. Rates could not be estimated at the health unit-level or per infecting *Plasmodium* species as travel data at this scale was not available.

Variable means (e.g. number of cases according to *Plasmodium* species) were analyzed by using one-way analysis of variance [17]. Tests for independence (e.g. to test if distribution of *Plasmodium* species was heterogeneous among health units) were performed with Chi-square analyses. Significant differences among means were determined by using Tukey’s method of multiple comparisons, with statistical tests considered significant at a family error rate of p<0.05 (Minitab 16; R). Raw data are presented in tables and figures; however, analyses were performed on transformed data (e.g. logarithmic transformations), where data did not display a normal distribution.

## Results

### Ontario Cases

**Figure 1:**
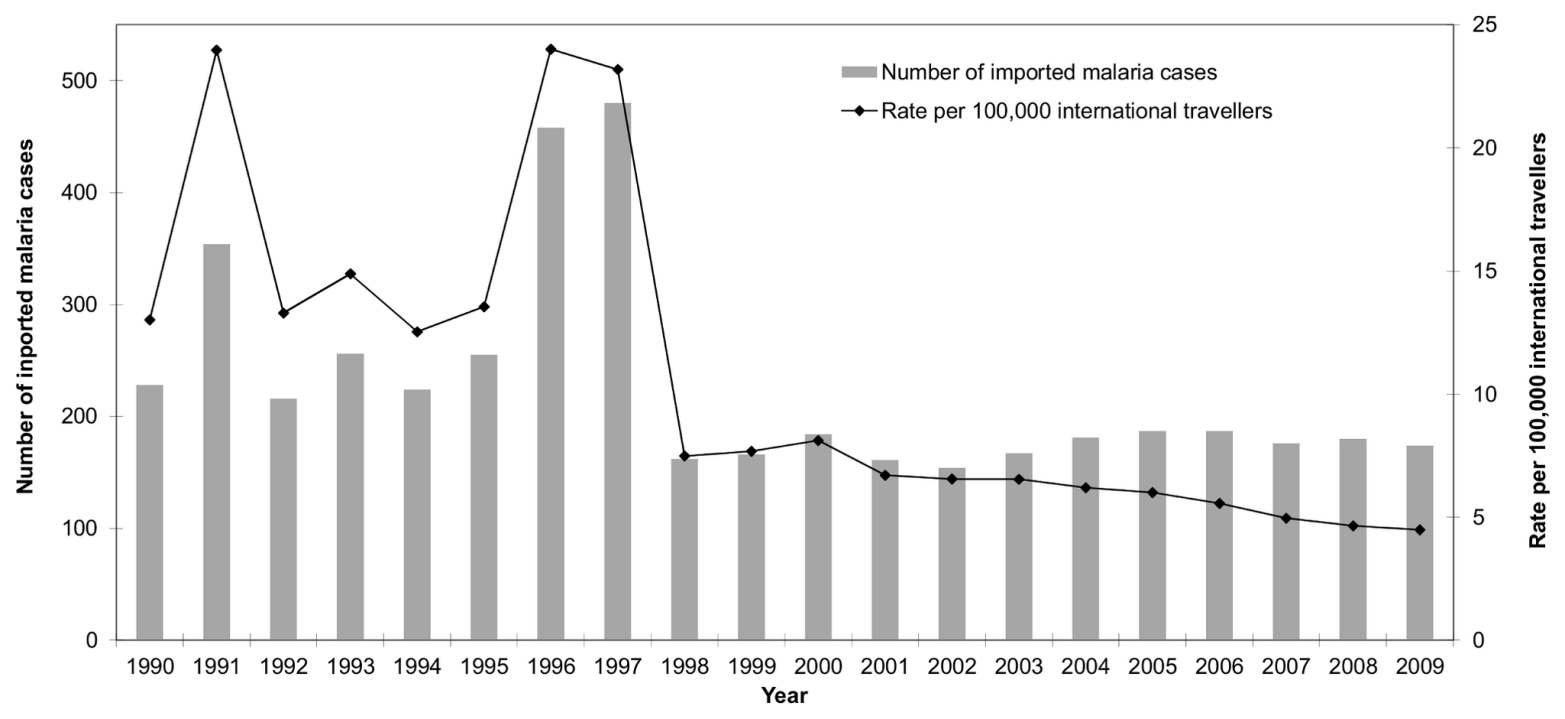
Imported malaria trends in Ontario, Canada (1990–2009) *, †, ‡. *Total number cases for each *Plasmodium* sp. and percentage of all cases: *P. vivax* (2305, 50.6%), *P. falciparum* (1751, 38.6%), *Plasmodium* sp. (272, 6.0%), *P. ovale* (142, 3.1%), and *P. malariae* (81, 1.8%). † Mean rate per 100,000 international travellers (for all *Plasmodium* species) for study period (1990–2009) was 10.7 ± 1.45; 15.4 ± 1.98 (1990–1999); 6.0 ± 0.35 (2000–2009). ‡Rate per 100,000 international travellers estimated based on total Canadians travelling to international locales (except USA) and returning to Ontario [13].

There were 4,551 confirmed cases of malaria reported in Ontario from 1990 through 2009 (mean 227.4 ± 22.38 cases per year; range 154-480; mean rate of infection = 10.7 ± 1.45 per 100,000 international travellers) (Figure 1). *Plasmodium vivax* was the most commonly reported infection during the reporting period with a mean of 115 ± 21.32 cases per year, which was not significantly more than the mean number of *P. falciparum* cases reported per year (87.6 ± 3.40) (Table 1). There were 13.5 ± 3.21 cases of *Plasmodium* sp. per year, followed by *P. ovale* (7.1 ± 0.99) and *P. malariae* (4.1 ± 0.49).

**Table 1:** Imported malaria cases by *Plasmodium* agent in Ontario, Canada (1990–2009).

**Species**	Mean ± SE malaria cases per year*			
	**Ontario (all health units)**	**Toronto**	**Peel**	**Ottawa**
*Plasmodium vivax*	115.3 ± 21.32a	53.9 ± 9.10a	30.8 ± 8.52a	5.8 ± 0.90b
*Plasmodium falciparum*	87.6 ± 3.40a	49.5 ± 2.51a	11.1 ± 1.12b	8.3 ± 0.89a
*Plasmodium ovale*	7.1 ± 0.99b	3.2 ± 0.67b	1.2 ± 0.25b	0.6 ± 0.15c
*Plasmodium malariae*	4.1 ± 0.49b	1.8 ± 0.35b	0.8 ± 0.22b	0.2 ± 0.09c
*Plasmodium* sp.	13.5 ± 3.21b	4.8 ± 1.68b	3.7 ± 1.30b	2.3 ± 0.52c
F_4,95_	28.4†	38.2†	10.5†	32.4†

* For each jurisdiction, means followed by different letters are significantly different at an individual error rate of p<0.01 (i.e. 0.05/5 *Plasmodium* species = 0.01); post hoc analysis of significant differences among means was performed with Tukey’s method of multiple comparisons. † Significant at p<0.0001.

**Figure 2:**
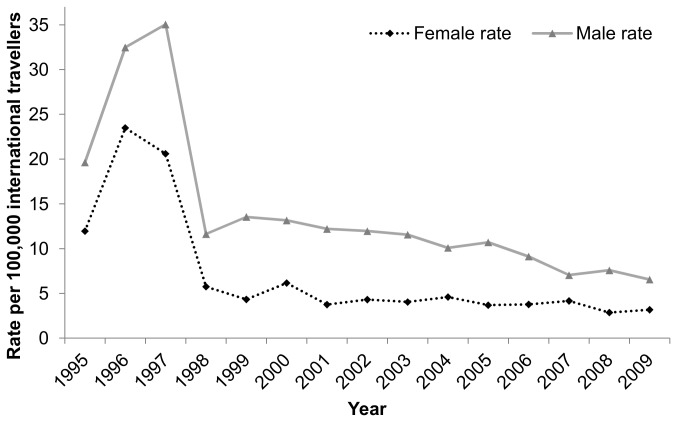
*Plasmodium* infection rates in male and female travellers for imported malaria cases (all *Plasmodium* species) in Ontario, Canada (1995–2009) *. *Rate per 100,000 international travellers estimated based on total Canadians returning from travel to international locales (except USA) and returning to Ontario [14].

Provincially, significantly more cases occurred per year in males than in females for all malarial species combined (Table 2). There were significantly more males infected per year with *P. falciparum* than females. There was no significant difference between the mean number of males and females infected per year with *P. vivax*. Frequency of male and female cases was dependent on health unit (*X* ^2^ = 14.3, p = 0.0007) and dependent on infecting *Plasmodium* species (*X* ^2^ = 41.3, p<0.0001). The rate of infection per 100,000 male travellers was significantly higher (approximately 2×) than the rate for female travellers (F_1,38_ = 12.1, p = 0.001) (Figure 2).

**Table 2:** Gender of imported malaria cases by jurisdiction and *Plasmodium* agent in Ontario, Canada (1990–2009).

**Case gender**	**Mean ± SE malaria cases per year**							
	**Ontario (all health units)**		**Toronto**		**Peel**		**Ottawa**	
Male	143.7 ± 11.29		72.4 ± 5.44		27.5 ± 4.27		11.4 ± 0.96	
Female	83.1 ± 10.06		40.0 ± 4.84		20.1 ± 4.24		5.9 ± 0.59	
F_1,38_	16.1, p<0.001		19.9, p<0.0001		1.5, p = 0.23		23.8, p<0.0001	
M:F*	1.73		1.81		1.37		1.93	
	***P. vivax***	***P. falciparum***		***P. ovale***		***P. malariae***		** *Plasmodium* sp.**
Male	68.8 ± 11.39	60.4 ± 2.83		4.0 ± 0.73		2.6 ± 0.32		8.0 ± 1.80
Female	46.0 ± 9.97	27.0 ± 1.46		3.1 ± 0.40		1.5 ± 0.29		5.5 ± 1.53
F_1,38_	2.3, p = 0.14	109.6, p<0.0001		1.2, p = 0.29		6.0, p = 0.019		1.1, p = 0.30
M:F*	1.50	2.24		1.29		1.73		1.45

* M:F, male: female ratios calculated using total male and female cases reported during entire study period for each jurisdiction or *Plasmodium* species.

**Figure 3:**
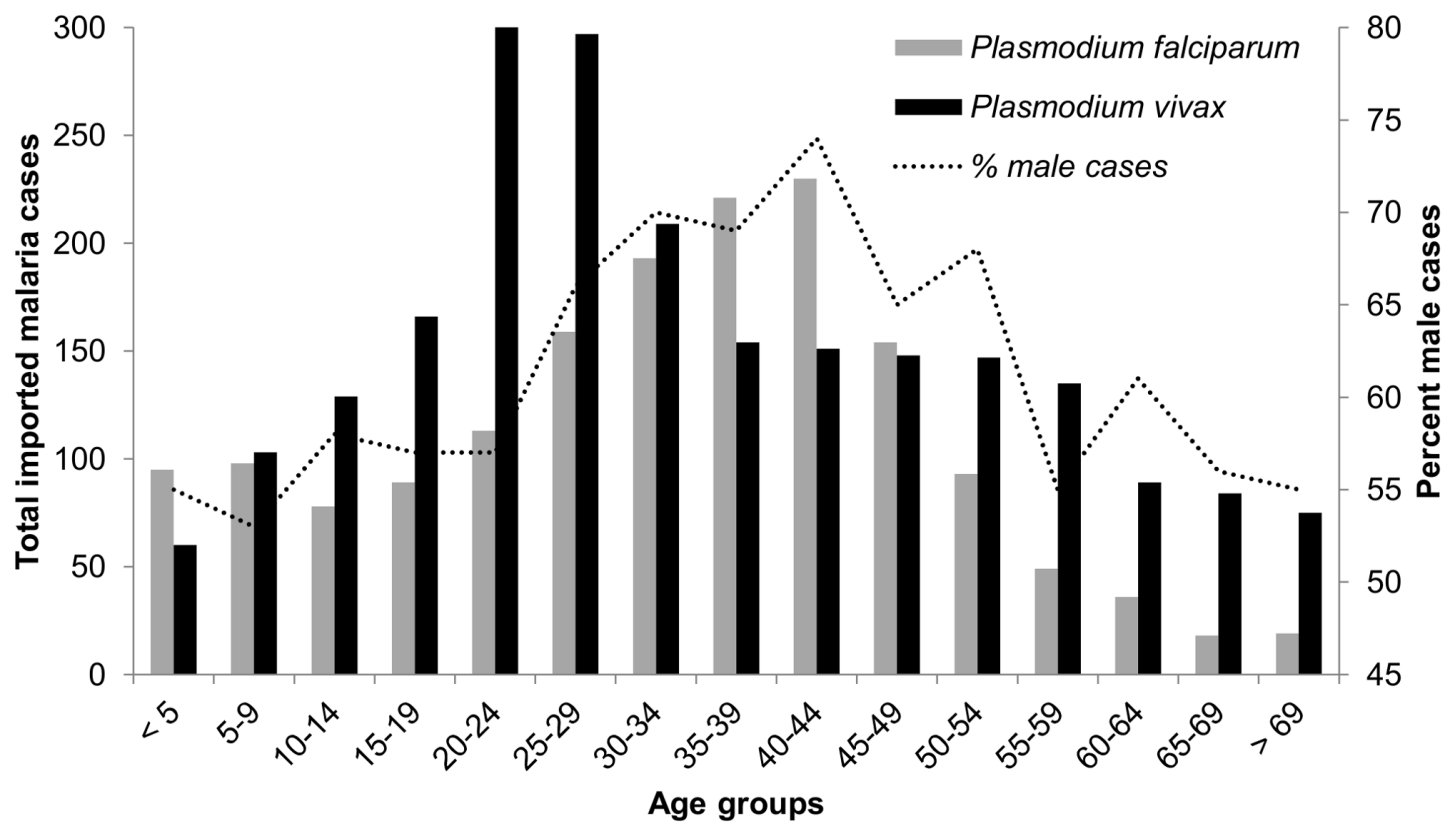
*Plasmodium falciparum* and *Plasmodium vivax* cases according to age at time of illness and percent male cases (all *Plasmodium* species) in Ontario, Canada (1990–2009) *. *For all *Plasmodium* species, the mean age at time of illness onset was 33.8 ± 0.26. There was no significant difference in mean age among *Plasmodium* species, *P. falciparum* (32.8 ± 0.39), *P. vivax* (34.0 ± 0.38), *P. malariae* (32.2 ± 2.25), and *P. ovale* (32.1 ± 1.27) (F_3,4275_ = 1.9, p = 0.13).

**Figure 4:**
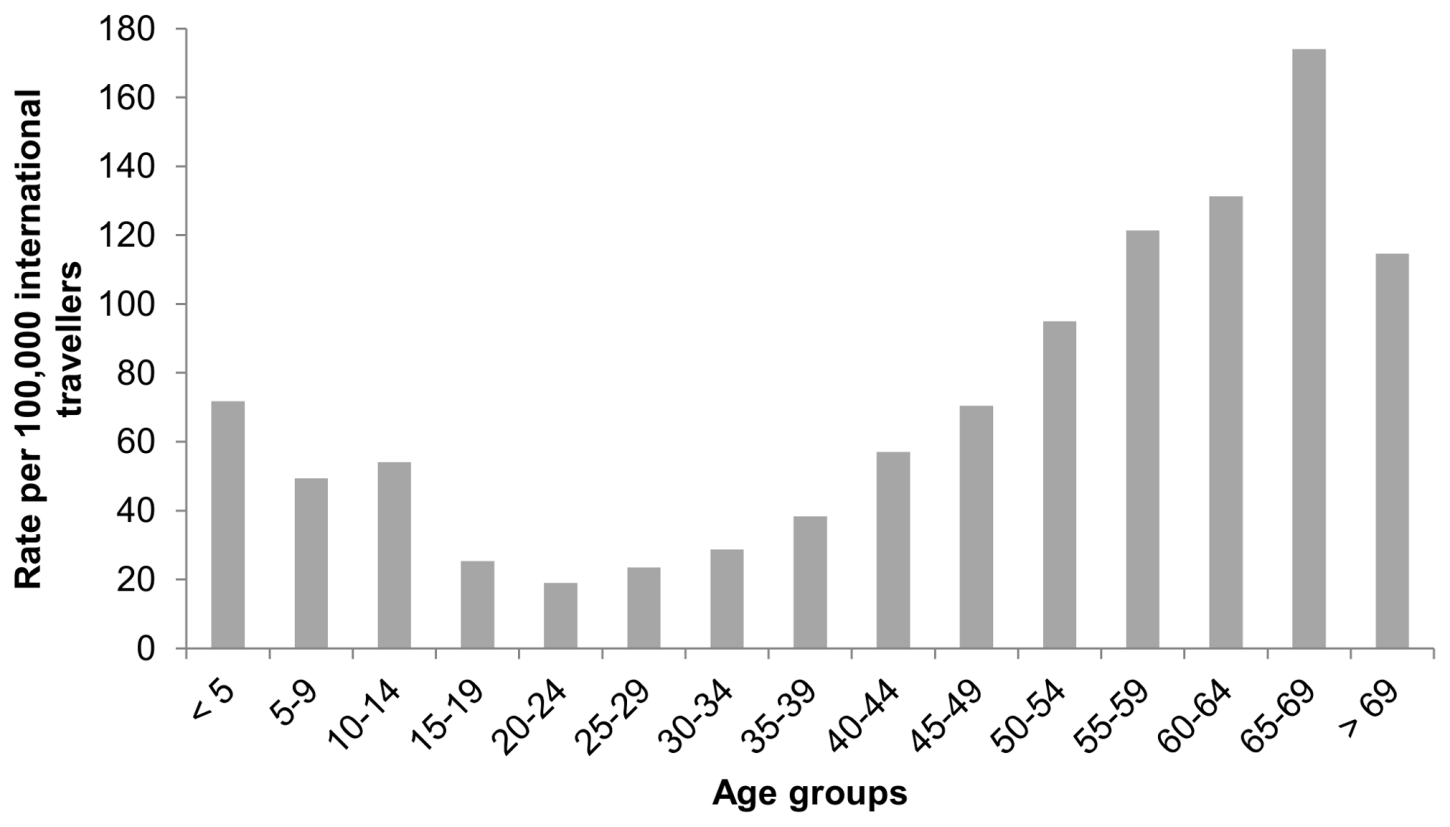
Rates of *Plasmodium* infection per age group for imported malaria cases in Ontario, Canada (1990–2009) *. *Rates per 100,000 international travellers estimated based on total non-permanent residents travelling to Ontario (1990–2009) [15].

For all *Plasmodium* species, mean age at time of illness onset was 33.8 ± 0.26 years old; however, there was no significant difference in mean age among patients infected with different *Plasmodium* species (Figure 3). Approximately 66% of all cases occurred in those 20–54 years old and 60% of female cases and 70% of male cases were part of this age range. The percent of all cases (all *Plasmodium* species) that were male is more than 50% in all age groups; however, the percentage was greatest in the 40–44 year old age group (74%) (Figure 3). In children less than 5 years old, the estimated rate of infection per 100,000 international travellers was approximately 70, decreasing to a low of 20 in 20–24 year olds and then steadily increasing to over 170 in 65–69 year olds (Figure 4).

**Figure 5:**
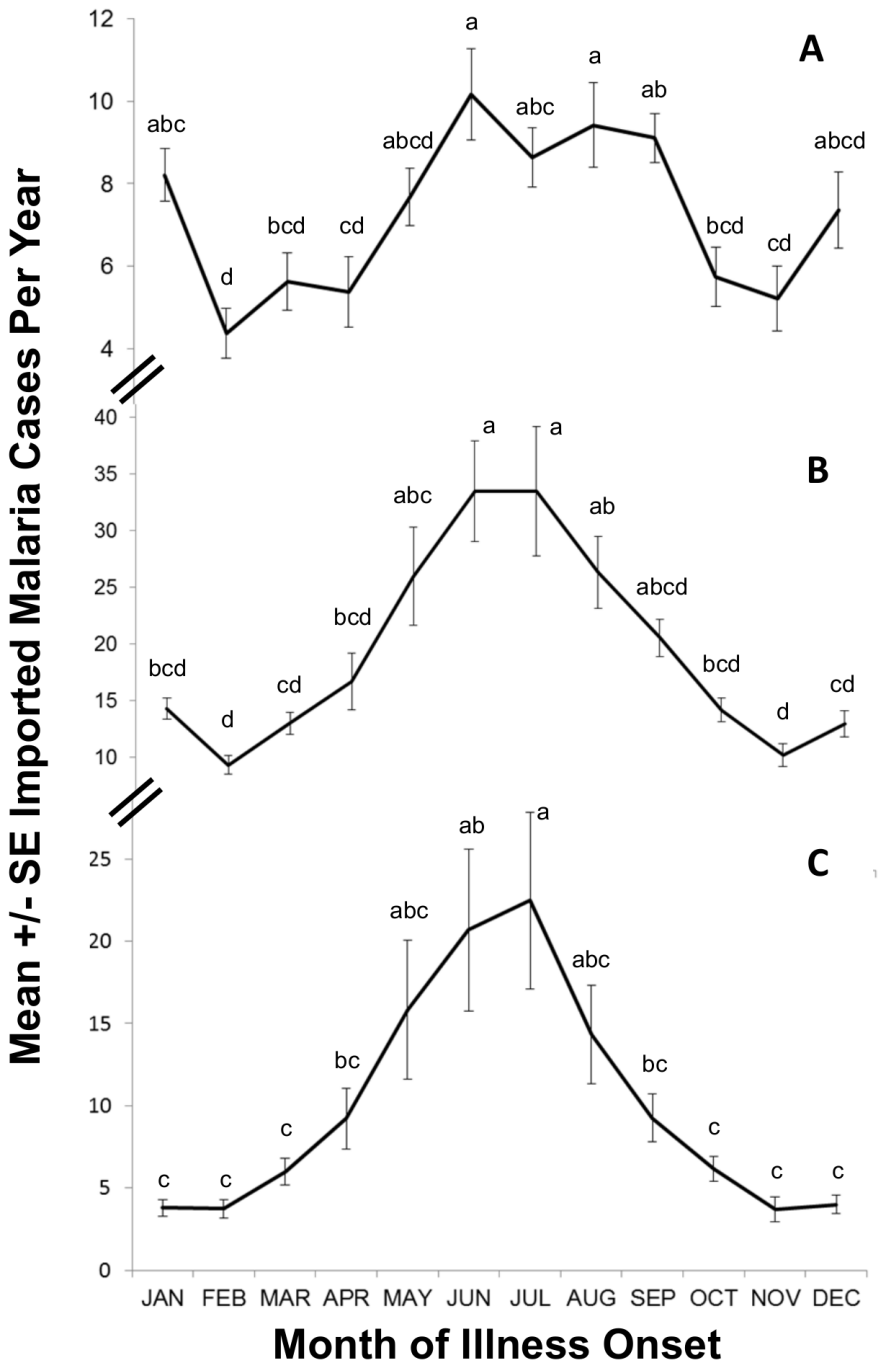
Phenology of imported malaria cases in Ontario, Canada (1990–2009) *. **A**. *Plasmodium falciparum*. **B**. All *Plasmodium* species. **C**. *Plasmodium vivax*. *ANOVA for A, F_11,228_ = 5.8, p<0.0001; B, F_11,228_ = 9.3, p<0.0001; C, F_11,228_ = 6.3, p<0.0001. For each chart, means that do not share a common letter are significantly different at an individual error rate of p<0.0042 (i.e. 0.05/12 months = 0.0042); post hoc analysis of significant differences among means was performed with Tukey’s method of multiple comparisons.

**Figure 6:**
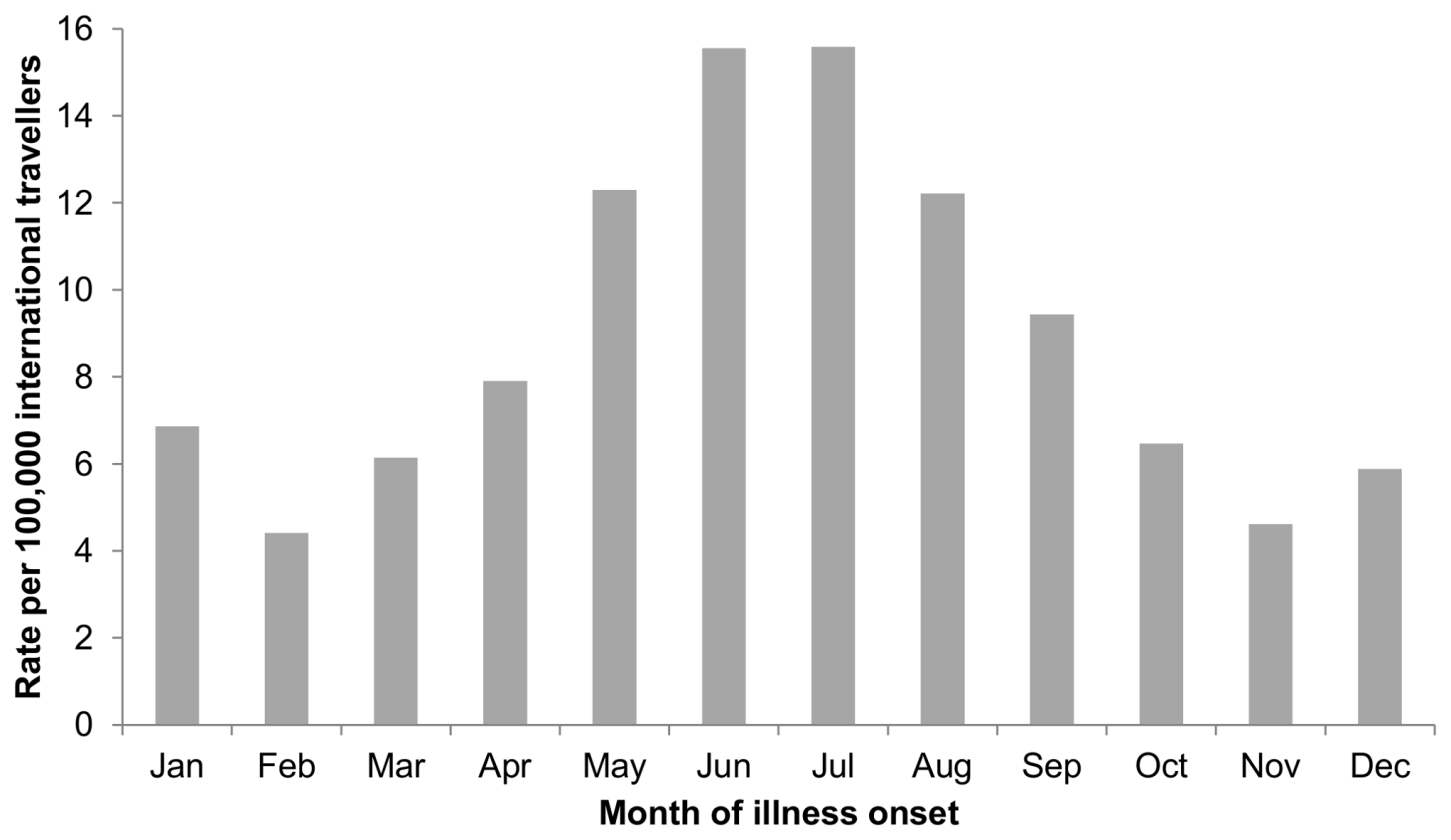
*Plasmodium* infection rate per month for imported malaria cases (all *Plasmodium* species) in Ontario, Canada (1990–2009) *. *Rate per 100,000 international travellers estimated based on total Canadians travelling to international locales (except USA) and returning to Ontario [13].

A peak in mean number of cases per year occurred in June and July for all *Plasmodium* species combined (Figure 5B). Seasonality was less evident for *P. falciparum* (increase in cases in June–August and a smaller peak in December–January) (Figure 5A); *P. vivax* seasonality mirrored the trend observed for all *Plasmodium* species with June and July peaks (Figure 5C). Estimated rates of *Plasmodium* infection per 100,000 international travellers peaked in June and July and, to a lesser extent, in December and January (Figure 6). The number of international travellers was not useful in predicting the monthly incidence of cases (R^2^ = 0.009; F_1,10_ = 0.1; p = 0.76).

Fifty countries were represented among the 409 cases where exposure country was reported (2006–2009), with 69% of cases reporting travel to or emigration from Nigeria (106 cases), Ghana (98), and India (78). Based on numbers of international travellers to certain regions of the world, estimates for rates of infection per 100,000 international travellers were calculated: Africa (226.0), Asia (6.1), Oceania and Australia (1.9), Americas (1.8), and Europe (0.1) (Table 3) [18]. Sixty-six percent of cases reported exposures in Africa, 27% from Asia, and the remaining 6% from other regions including Oceania and the Americas (Table 3). Seventy-one percent of *P. falciparum* cases reported exposure in Ghana or Nigeria and 63% of *P. vivax* cases reported exposure in India.

**Table 3:** Exposure regions for imported malaria cases in Ontario, Canada (2006–2009).

Exposure region*,†	**Malaria cases** (2006–2009)					**Total (%)**	**Estimated rate per 100,000 travellers‡**
	***P. falciparum***	***P. vivax***	***P. ovale***	***P. malariae***	** *Plasmodium* sp.**		
Africa	229	9	12	5	15	**270 (66.0)**	**226.0**
Asia	11	86	4	1	9	**111 (27.1)**	**6.1**
Americas	8	8	0	0	6	**22 (5.4)**	**1.8**
Oceania and Australia	1	1	0	0	2	**4 (1.0)**	**1.9**
Europe	2	0	0	0	0	**2 (<1)**	**0.1**
**Total**	**251**	**104**	**16**	**6**	**32**	**409 (100)**	

* Exposure region: country. **Africa**: Angola, Botswana, Cameroon, Central African Republic, Chad, Côte d’Ivoire, Democratic Republic of the Congo, Ethiopia, Gabon, Ghana, Guinea, Kenya, Liberia, Madagascar, Mali, Niger, Nigeria, Sierra Leone, South Africa, Sudan, Tanzania, Uganda, and Zambia; **Asia**: Bangladesh, China, India, Indonesia, Myanmar, Pakistan, South Korea, Sri Lanka, and Thailand; **Americas**: British Virgin Islands, Cayman Islands, Cost Rica, Cuba, Dominican Republic, Guyana, Haiti, Honduras, Jamaica, Mexico, Nicaragua, and USA; **Oceania and Australia**: Australia, Papua New Guinea, and Tuvalu; **Europe**: Portugal and Spain.

† Exposures reported from malaria-free countries were clarified when further examined, such as Portugal (travel to Africa), 1 of 2 cases from Tuvalu (travel to Uganda), and USA (travel to Côte d’Ivoire). However, several cases remain cryptic, (i.e. likely exposure is a malaria-free country): Australia, British Virgin Islands, Cayman Islands, Cuba, Spain, and 1 of 2 cases in Tuvalu [18].

‡ Rates per 100,000 international travellers estimated based on total number of non-permanent residents travelling to Ontario from a particular region (2006–2009) [16].

**Figure 7:**
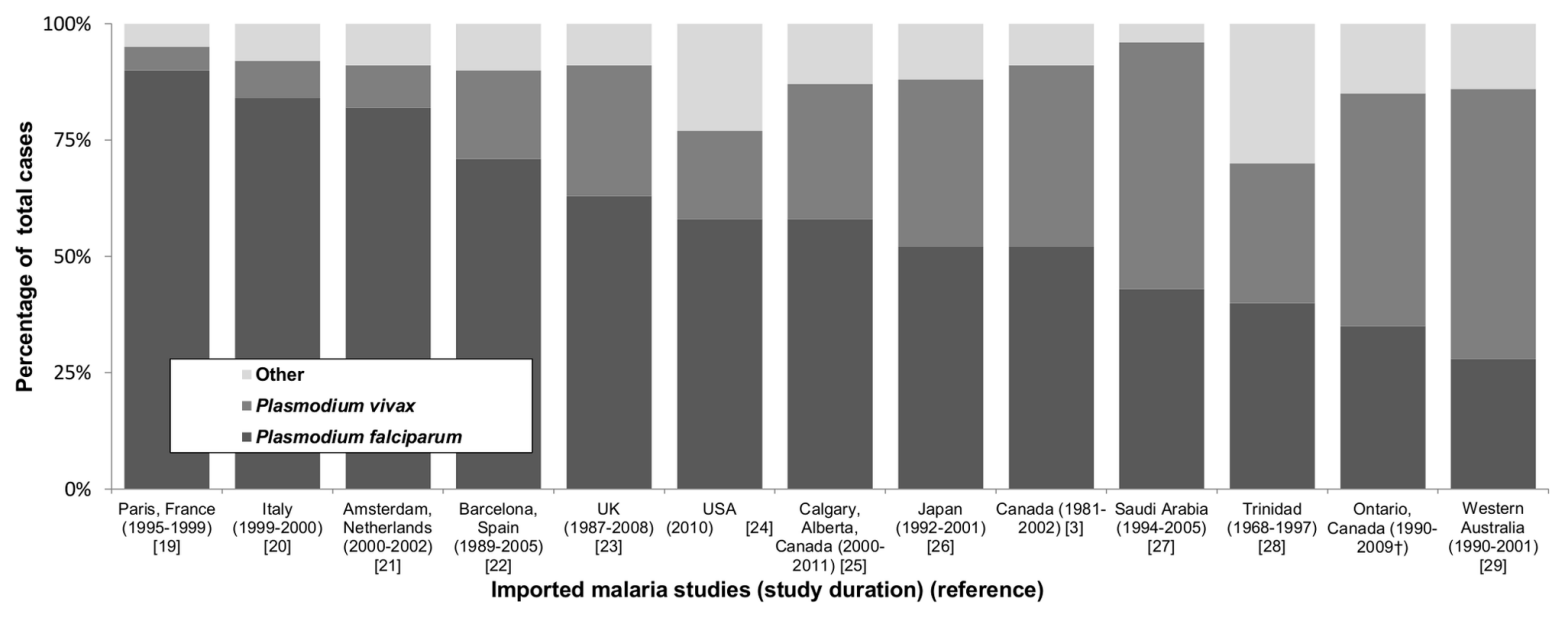
Percentage of imported malaria cases caused by *Plasmodium falciparum, Plasmodium vivax*, and “Other” (e.g., *Plasmodium ovale*, *Plasmodium malariae*, unknown, and unspecified or mixed) according to jurisdiction*. *For period **1990–2009**: *P. vivax* (51%), *P. falciparum* (39%), and “Other” (10%). Not shown for **1990–1999**: *P. vivax* (64%), *P. falciparum* (28%), and “Other” (10%). Not shown for **2000–2009**: *P. vivax* (30%), *P. falciparum* (55%), and “Other” (15%). † This study.

We compared the percentage of cases caused by *P. falciparum* and *P. vivax* in various jurisdictions, including Ontario (Figure 7) [3,19–29]. *Plasmodium falciparum* was the principal etiological agent reported in Paris, France (90% of all cases) compared with Western Australia where only 24% of all cases are due to *P. falciparum*. In Ontario, 39% of malaria cases were caused by *P. falciparum* (1990–2009). Although *P. vivax* dominated cases for the entire study period (and from 1990–1999), there were more *P. falciparum* cases compared to *P. vivax* cases from 2000 through 2009 (Figure 7; see footnote).

### Local Cases

**Figure 8:**
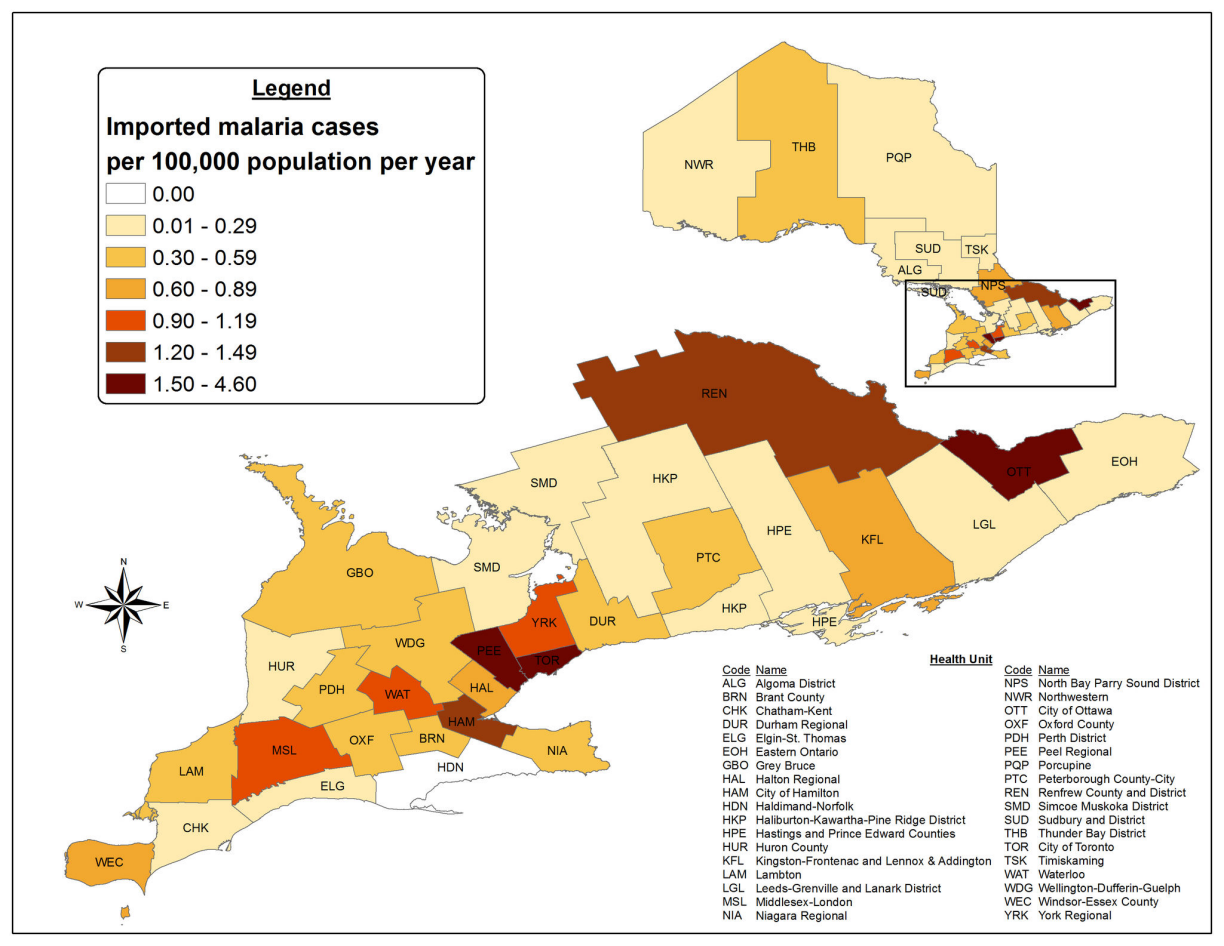
Imported malaria distribution and case rate in Ontario’s health units (1990–2009).

Thirty-five out of Ontario’s 36 health units reported at least one imported malaria case during the surveillance period (Figure 8). Toronto, Peel, and Ottawa reported 2,260 (rate, 1.9 cases per 100,000 health unit population); 951 (rate, 3.9); and 344 (rate, 2.2) cases, respectively. Collectively, these three health units reported 78% of all cases in Ontario; including 79% of all *P. falciparum* cases and 79% of all *P. vivax* cases (these three health units represent 33.3% of Ontario’s population). In Peel, there were a significantly higher mean number of cases per year of *P. vivax* than *P. falciparum* (Table 1). In Ottawa, there were a significantly higher mean number of cases of falciparum than vivax malaria. In Toronto, there was no significant difference between mean number of cases per year caused by *P. falciparum* and *P. vivax*. In Toronto and Ottawa, significantly more cases were reported per year in men than women; however, no significant gender differences were noted in Peel (Table 2). The frequency of *Plasmodium* species was dependent on health unit (X^2^ = 190.1, p<0.0001).

## Discussion

We report 4,551 cases of imported malaria and an average infection rate of 10.7 per 100,000 international travellers during the 20-year surveillance period. The incidence of cases has remained stable since 1998; however, with increasing international travel, the rates of infection have been decreasing since 2000. The increase in cases in 1996 and 1997 has been noted previously in other non-malaria-endemic jurisdictions (i.e., British Columbia, Canada; UK) and, while exposure information for Ontario cases is lacking for these years, the increase is likely due to malaria epidemics in several northwestern states of India such as Haryana and Rajasthan [3,23,30]. In 2009, the only sub-national jurisdiction in Canada or the USA with a greater number of cases than Ontario (174 cases) was New York State, USA (265 cases) [24]. The total number of imported malaria cases, as with other reportable diseases, is underestimated in passive surveillance systems (i.e. 20–59% of cases missed); therefore, the true incidence of imported malaria in Ontario is expected to be greater than we report [31] Opportunities to decrease the incidence of imported malaria in Ontario include continued and focused education of international travellers of the risks of *Plasmodium* infection and renewed attention to established guidelines for Canadian travellers to malaria-endemic regions [32].

In Ontario, *P. vivax* was the dominant malarial agent during the first half of the surveillance period (1990–1999); however, since 2000, *P. falciparum* has supplanted *P. vivax* as the dominant agent. An increase in *P. falciparum* cases and associated decrease in *P. vivax* was similarly noted in a UK study (1987–2006) [23]. The increase in *P. falciparum* cases in the UK was attributed to increased travel to West Africa, a region with high *P. falciparum*-transmission rates. The purpose or reason for travel to West Africa was not captured in our passive surveillance systems; however, jurisdictions with similar imported malaria etiology (i.e., UK; Amsterdam, The Netherlands) have identified that most travellers to West Africa are classified as visiting friends and relatives (VFRs) [21,23]. Travellers identified as VFRs are at higher risk for *Plasmodium* infection due to several factors, including 1) visiting malaria-endemic countries for longer periods, 2) travel to areas of higher transmission intensity, 3) belief that they are immune, and 4) a lack appropriate pre-travel advice [21–23]. The *P. vivax* decrease trend in the UK was attributed to an increase in the proportion of travellers to South Asia that visited urban areas (putatively, cities possess more effective vector-management programs compared to rural locales; therefore, a decreased risk of *P. vivax* infections) [23]. The reasons for the change in imported malaria epidemiology in Ontario are not known, but likely involves changes within Ontario (e.g., demographics, international travel patterns) and changes in malaria-endemic regions (e.g., socioeconomic and political factors impacting vector-control and prevention programs) that consequently increased the incidence of falciparum malaria and decreased the incidence of vivax malaria.

Distribution of *Plasmodium* species in Ontario was associated with diagnosing health unit. Patterns seen in a jurisdiction’s *Plasmodium* composition mirror its population’s indigenous background, language, and colonial ties to malaria-endemic countries. For example, France’s population maintains a close relationship to former French colonies, with a greater number of its cases having originated in Cameroon and Côte d’Ivoire [33]. In the UK, malaria cases originated in English-speaking Ghana and Nigeria and the former colony of India, approximating the results identified for Ontario [23]. Ontario’s immigrant population originates from Asia (40%), followed by Europe (39%), Central/South America and Caribbean (13%), Africa (5%), and other regions (3%) [34]; however, immigrant composition varies by health unit, leading to variation in the prevalence of *Plasmodium* species. Relative to provincial percentages, Peel has a greater percentage (44%) of Asian-born residents and a greater prevalence of *P. vivax* [34]. In contrast, Toronto has a greater percentage of African immigrants (6%) and a greater prevalence of *P. falciparum*. One limitation to our study is that travel data for patients at the health unit level were not available, but further investigation of prospective cases (with travel history) will provide further insight into risk or rate of infection for travellers at this scale. In recent research, Ontario’s imported malaria cases clustered at the neighborhood level, with clustering particularly evident in low-income areas [35]. The indigenous background of residents, at the local level in Ontario, dictates travel patterns and subsequent infection by specific *Plasmodium* species; therefore, tailoring pre-travel education (considering patient language, culture, and access to resources) at the local level should prove beneficial to higher-risk populations.

Men accounted for the majority of imported malaria cases in Ontario and most of cases occurred in those 20–54 years old; however, estimated rates of infection per 100,000 international travellers were greatest in those over 50 years old. In Ontario, females make up the majority of international travellers (57%), yet our analysis indicated that the rate of *Plasmodium* infection in males was twice that of females [14]. Predominance of men in imported malaria is well represented in the literature, for example Western Australia (80% of all cases are men); Amsterdam, the Netherlands (69%); and the UK (62%) are jurisdictions with a disproportionate number of male cases [21,23,29]. The distribution of male and female cases was not only associated with the diagnosing health unit, but also associated with infecting *Plasmodium* species. Toronto and Ottawa had a relatively higher male to female case ratio and a greater percentage of the province’s *P. falciparum* infections; however, Peel had a relatively lower male to female case ratio and a greater percentage of the province’s *P. vivax* infections. Infection rates vary by gender in malaria-endemic regions, where cultural traditions and occupation contribute to a gender disparity, providing a basis for the gender-specific patterns seen for imported malaria. Risk of infection is often greater in men because they are more likely to migrate to malaria-endemic areas for agricultural or mining work and more likely to sleep outdoors during peak biting times of vectors; however, gender-specific behaviours of Ontario cases are unknown [36,37]. The mean age at time of illness (~34 years old for all *Plasmodium* species) in Ontario is similar to other jurisdictions such as the UK (31 years old), Calgary, Alberta (32 years old), and Barcelona, Spain (33 years old) [22,23,25]. Without reporting the estimated rate of infection among the age groups, the highest risk would appear to be in 20–54 year olds (based on incidence alone); however, estimating rates of infection for each age group provides a more robust assessment of risk, where those under 15 and those over 50 years old are at relatively higher risk. The underlying factors governing the dominance of male cases represents an area requiring further research, research that will provide a better understanding of the gender disparity in imported malaria cases.

Imported malaria cases in Ontario occurred more often in the summer (e.g., *P. falciparum*, June–August and, to a lesser extent, December–January; *P. vivax*, June–July). For all *Plasmodium* species, rates of infection were approximately 2.3-3.5× higher in June and July compared to October through March. The number of international travellers was not useful in explaining the monthly incidence of cases in Ontario, revealing that risk of infection is not based solely on volume of travellers to endemic regions, rather that other factors contribute more to risk (i.e., travel destination). Similarly, in the UK, P. *falciparum* cases peaked in September and again in January; *P. vivax* cases peaked in July [23]. For *P. vivax*, case increases are because of greater travel volume in the summer, a period when there is also increased risk in destination countries (e.g. summer monsoon season in India). Pre-travel health care providers consider seasonality (dry versus rainy season of destination), as a part of an overall exposure assessment prior to a patient’s travel [32]. The phenology described here for imported malaria will help define periods of increased risk of infection, for Ontario travellers adding to a more informative exposure assessment prior to travel.

More than 50% of all imported malaria cases in Ontario, where country of exposure was reported (2006–2009), originated in Ghana or Nigeria and more than 19% of all cases originated in India. Travel to Ghana and Nigeria accounted for 34% of all cases in the UK [23], 32% of cases in Trinidad [28], and 24% of cases in the USA [24]. Travel to India accounted for 21% of cases in Trinidad [28] and 6% of cases in the USA [24]. When assessing risk for travellers to particular regions, rates of infection were 37× higher in travellers from Africa (226.0 per 100,000 travellers) compared to travellers from Asia (6.1) and 126× higher compared to travellers from the Americas (1.8). A limitation to our study is that exposure data was missing for approximately 43% of the cases (2006–2009); however, there is no evidence to suggest that cases with missing country of exposure information are appreciably different from the exposure trends reported here. Further, estimates of rates of infection per 100,000 international travellers are based on total travellers from each region and not all travellers from a region are at risk of *Plasmodium* infection (e.g. travellers from Africa include those travelling from high-risk countries such as Nigeria compared to those from low-risk countries like Egypt); therefore, our estimates for rates of infection are likely lower than the true rates for travellers to malaria-endemic countries [18]. While relatively high, our estimated rate for Africa (226 infections per 100,000 international travellers) is reasonable given rates for European travellers to Kenya can range from 18 to 207 per 100,000 travellers (depending on European country of residence) [38]. Where demographics and temporal dynamics contribute to the overall risk for travellers, travel destination contributes disproportionately to this risk, indicating that pre-travel programs tailored for travellers to West Africa and India are essential.

Travel destination for Ontario travellers represents the main risk factor for acquiring *Plasmodium* abroad; however, assessing risk should consider health unit-, spatiotemporal-, and demographic-specific dynamics. Despite being a rare event in North America, local transmission of *Plasmodium* will continue to occur, leaving surveillance as an important component of protecting public health [8,39]. Surveillance was vital to the detection and management of recent outbreaks of *P. vivax* malaria in Greece where locally-acquired infections were epidemiologically linked to imported cases [40]. Surveillance data and, more importantly its analysis, are essential in identifying factors that underlie the evolving epidemiology of imported malaria and for developing public health programs (i.e. public education). Malaria’s reach, from the world’s endemic regions to the cosmopolitan centers of Ontario, will continue to shorten; nevertheless, a better understanding of risk will aid in the development of targeted, pre-travel programs.
